# Development and Evaluation of Treatment Adherence Interventions for Older Adults With MCI Using IoT Devices

**DOI:** 10.1093/geroni/igab046.2600

**Published:** 2021-12-17

**Authors:** Jinhee Shin, Eunhee Cho, Gwang Suk Kim, Heejung Kim, Byoung Seok Ye, Chang-Gi Pack

**Affiliations:** 1 Yonsei University College of Nursing, Seoul, Seoul-t'ukpyolsi, Republic of Korea; 2 Yonsei University, Seoul, Seoul-t'ukpyolsi, Republic of Korea; 3 University of Illinois at Chicago, Seoul, Illinois, United States

## Abstract

For older adults with mild cognitive impairment (MCI), treatment adherence is essential to prevent and delay dementia. Older adults with MCI should maintain treatment for chronic diseases, exercise regularly, and adhere to treatment to maintain health status. There is a lack of comprehensive interventions to promote treatment adherence (medication adherence and physical activity) for older adults with MCI. The purpose of this study was to develop an internet of things (IoT)-based real-time treatment adherence for old adults with MCI and examine the effectiveness of the program. This study was a randomized controlled trial. The patients were enrolled from the neurology outpatient department clinic at a hospital in Korea. The subjects were 18 in the experimental group and 20 in the control group. This study intervention was IoT-based medication adherence device and real-time monitoring sever plus wrist wearable device. The study consists of a 10-week intervention period. The intervention program was provided for only the experimental group and the control group with a wearable device and usual care. Assessments were conducted at baseline, 6 weeks, and 10-weeks. A mixed-effects model was used in the analysis to evaluate the program. The IoT-based treatment adherence intervention was effective in improving medication adherence over time (β =11.465, p<.001), physical activity (K-PASE) (β =27.376, p<.001) and average the number of steps per week (β=3202.53, p<.001). Health care providers can use this program to improve treatment adherence for chronic disease management and dementia prevention of older adults with cognitive impairment.

